# Validation of Housekeeping Genes as Reference for Reverse-Transcription-qPCR Analysis in Busulfan-Injured Microvascular Endothelial Cells

**DOI:** 10.1155/2018/4953806

**Published:** 2018-10-02

**Authors:** Wen Ju, Alhaji Osman Smith, Tiantian Sun, Pingping Zhao, Yan Jiang, Lu Liu, Ting Zhang, Kunming Qi, Jianlin Qiao, Kailin Xu, Lingyu Zeng

**Affiliations:** ^1^Key Laboratory of Bone Marrow Stem Cell, Jiangsu Province, Xuzhou 221002, China; ^2^Department of Hematology, The Affiliated Hospital of Xuzhou Medical University, Xuzhou 221002, China; ^3^Blood Diseases Institute, Xuzhou Medical University, Xuzhou 221002, China

## Abstract

Endothelial cells (ECs) could express some important cytokines and signal molecules which play a key role in normal hematopoiesis and repopulation. Busulfan-induced vascular endothelial injury is an important feature after hematopoietic stem cell transplantation (HSCT). But the molecular mechanism of how the injured ECs affect hematopoietic reconstruction is still unknown. It is possibly through modulation of the change of some gene expression. RT-qPCR is one of the most popular methods used to accurately determine gene expression levels, based on stable reference gene (RG) selection from housekeeping genes. So our aim is to select stable RGs for more accurate measures of mRNA levels during Busulfan-induced vascular endothelial injury. In this study, 14 RGs were selected to investigate their expression stability in ECs during 72 hours of EC injury treated with Busulfan. Our results revealed extreme variation in RG stability compared by five statistical algorithms. ywhaz and alas1 were recognized as the two idlest RGs on account of the final ranking, while the two most usually used RGs (gapdh and actb) were not the most stable RGs. Next, these data were verified by testing signalling pathway genes ctnnb1, robo4, and notch1 based on the above four genes ywha, alas1, gapdh, and actb. It shows that the normalization of mRNA expression data using unstable RGs greatly affects gene fold change, which means the reliability of the biological conclusions is questionable. Based on the best RGs used, we also found that robo4 is significantly overexpressed in Busulfan-impaired ECs. In conclusion, our data reaffirms the importance of RGs selection for the valid analysis of gene expression in Busulfan-impaired ECs. And it also provides very useful guidance and basis for more accurate differential expression gene screening and future expanding biomolecule study of different drugs such as cyclophosphamide and fludarabine-injured ECs.

## 1. Introduction

The vascular endothelial cells (ECs) are particularly vulnerable to toxic effect of preparative regimen drugs, such as Busulfan, cyclophosphamide, and fludarabine which are widely used prior to hematopoietic stem cell transplantation (HSCT) [1]. Several studies have indicated that bone marrow (BM) vascular niche was impaired after HSCT [2–5], which was associated with poor Graft Function [3, 4]. The healthy ECs, their expressed cytokines, and signal molecules in BM microenvironment play an important role in normal hematopoiesis and repopulation [6–8], while the function of the impaired ECs, the changes of expressed cytokines and signal molecules, and how do these changes affect hematopoietic cell function are still unknown. Because our and other previous studies [9, 10] found that ECs are essential to accelerate hematopoietic and immune reconstitution, we speculate that the occurrence of poor Graft Function is most likely related to abnormalities of preparative regimen-injured ECs and their gene expression change.

Busulfan, most widely used in HSCT, has been identified as having potent antitumor activity and inhibitory functions on normal hematopoiesis as well as myelogenous proliferation [11]. Most importantly, our study has shown that pretreatment with Busulfan for HSCT could induce obvious injury to ECs in vivo [2] but we still do not know the biomolecular mechanism. Therefore, in vitro studies of the biomolecular changes on normal and injured endothelial cells need to be clarified firstly, which is important for study on how the impaired ECs control HSC fate in the future.

Reverse-transcription-qPCR is one of the most widely used methods directly evolved from the end-point detection PCR to detect gene expression level under different research conditions because of its time-saving, high sensitivity, and specificity [12–14]. But if this technique is performed in an inappropriate way, especially using incorrect housekeeping genes (HKGs), considerable misinterpretation of results will happen [15]. The HKGs such as actb and gapdh which are found in different cells or tissues, known to maintain cellular functions, are the most widely used RGs. However, their stability varies under different experimental conditions [16, 17]. Moreover, several studies had reported that there is no single reference gene that can maintain its expression level in different experimental conditions [18–20]. Typically, internal control genes show variability in expression levels in different tissues, emphasizing the importance of identification for normalization reference validation selection.

For the biomolecule study of Busulfan on EC injury, identifying the most stable RGs in Busulfan-impaired EC system firstly is of great importance. But, based on our knowledge, stable HKGs selection in the damaged ECs has never been performed. So the purpose of this research is to recognize the most suitable HKGs in impaired ECs, which can be used as reference genes for normalization of qPCR results.

In the this study we used three software types including geNorm, NormFinder, and BestKeeper together with the delta-delta method [21–24] and Comprehensive Ranking methods [15, 25] to identify the most suitable RGs from 14 commonly used HKGs in both normal and impaired ECs. This study revealed the importance of RGs selection for the valid and reproducible analysis of gene expression in Busulfan-impaired ECs. And it also provides a very useful guidance and basis for more accurate differential expression gene screening and future expanding gene expression and biomolecule function study of different drugs such as cyclophosphamide and fludarabine-injured ECs.

## 2. Methods and Materials

### 2.1. Cultivation of Cell

The endothelial cells (bEnd.3) were purchased from the Global Bioresource Center of American Type Culture Collection (ATCC) and cultured in a medium which comprised DMEM and 10% FBS and then incubated at 37°C in incubator and humidified at 5% CO_2_, respectively. The medium containing DMEM, 10% FBS, and 30mg/L of Busulfan (purchased from Sigma-Aldrich) was added to the ECs and then observed at different time interval at 0, 12, 24, 36, 48, and 72 hours, respectively. All our samples were independently prepared three times.

### 2.2. Isolation of RNA

RNA was isolated from the normal and Busulfan-injured ECs using TRIZOL Reagent, Invitrogen kit according to the manufacturer's instructions [26]. The concentration and quality of the extracted RNA were calculated by NanoDrop 2000 spectrophotometer (Thermo, USA). The purity was confirmed by using the absorption ratio which was between 1.8 and 2. The integrity was also evaluated using 0.7% ~ 1.0% agarose gel electrophoresis indicating no contamination of the DNA and degradation of RNA. Genomic DNA contamination was determined by performing qPCR with total RNA as the template; no PCR product appearing on gel proved that no genomic DNA contaminated the total RNA.

### 2.3. Complementary DNA (cDNA) Synthesis

The intact RNA was reverse transcriptase at once after isolation using Invitrogen reagent M-MLV. Firstly, the RNA was transcribed into first cDNA, using 10*μ*m oligonucleotide dT primer; 10mM dNTP and DEPC-treated water were combined together and preserved at 65°C for 10 minutes with extended temperature of 4°C in the conventional PCR. The transcription mixture of 0.1M DTT, 50,000U M-mlv, and 5x-strand buffer was then incubated at 37°C for 50 minutes, at 70°C for 5 minutes and extended temperature of 4°C. All cDNA was synthesized from 300ng isolated RNA sample in a total volume of 20*μ*L and kept at -20°C until ready for use. All samples were diluted by 1:10 with DEPC water, in order to achieve equal concentration of our samples for RT-qPCR analysis.

### 2.4. RT-qPCR

The 14 candidate reference genes primers and three target genes (ctnnb1, robo4, and notch1) were designed and purchased from Thermo Scientific and were selected in consideration of different intracellular biological function as shown in Table 1. Primer sequence and information are also shown in Table 1. RT-qPCR was performed using Light Cycler®480 in multiwall plate 96. Each 20 *μ*L reaction contained 10*μ*L of 2× Supermix SYBR Green I Master, 1*μ*L forward and reversed primers, 8*μ*L of distilled water, and 1*μ*L of cDNA. The RT-qPCR program was comprised of predenaturation at 95°C for 1 minute, 40 cycles at 95°C for 20 seconds, 60°C for 15 seconds, and 72°C for 15 seconds.

The specificity of the primers and the size of the PCR products were checked using 2% agarose gel electrophoresis and gel-red staining. The threshold values (Cp value) were obtained using a fluorescence threshold of 1.0. Then the results were copied into input file, based on the software requirement. The efficiency of each primer was calculated using the standard gradient [%E = 10^(−1/A)^*∗*100] of RT-qPCR. The standard curve was obtained by plotting the Cp value (y axis) against the logarithm of the total cDNA concentration (x axis).

### 2.5. Statistical Analysis

The three excel based software types geNorm, NormFinder, and BestKeeper together with the comparative delta-delta Cp method were used to assess the selected RGs stability. Applying the geNorm software, the M value is used to assess the stability of the internal control gene and it was determined by stepwise removal of gene with higher M value. Based on this software, the reference gene with the lowest M value is considered the most stable, while those with higher values indicate least stability. In addition, according to [24], the use of maximum number of genes for normalization based on average pairwise variation of all the reference genes was proposed. The NormFinder software uses mathematical ANOVA, to estimate the stability of the reference gene; like the geNorm software gene with the lowest M value is considered the most stable [23]. The BestKeeper software calculates the standard deviation (SD), coefficient of variation (CV%), and Pearson correlation coefficient (r), respectively. These calculated values are then used to assess the stability of the selected reference gene. In this study, we use the Pearson coefficient (r= 0.936-0.977) to rank the stability of the selected gene [22].

The 2^-ΔΔCT^ method [21] is the easiest method that is used to detect the expression level of genes from RT-qPCR experiments. Using the ΔΔCt method the following steps are observed: Firstly: normalization of reference gene or lower Ct value {ΔCt = ΔCt (max) -ΔCt (mini)}; secondly: the lower ΔCT value is used as the control—then ΔΔCt is obtained by ΔCt_control_-ΔCt_reference_; the exponential expression is calculated by using ΔCt expression = 2^∧^-ΔΔCt; and thirdly: average replicates and calculating the SD, variance, and CV%. In this study we used the delta-delta Ct method and in order for us to obtain the true fold difference we take the log base 2 of the mean expressed value to even out the scale of up- and downregulated gene. Downregulated gene has a scale of 0-1, while upregulated gene has scale of 1-infinity.

## 3. Results 

### 3.1. Primer Efficiency and Specificity Detection

**Figure 1:**
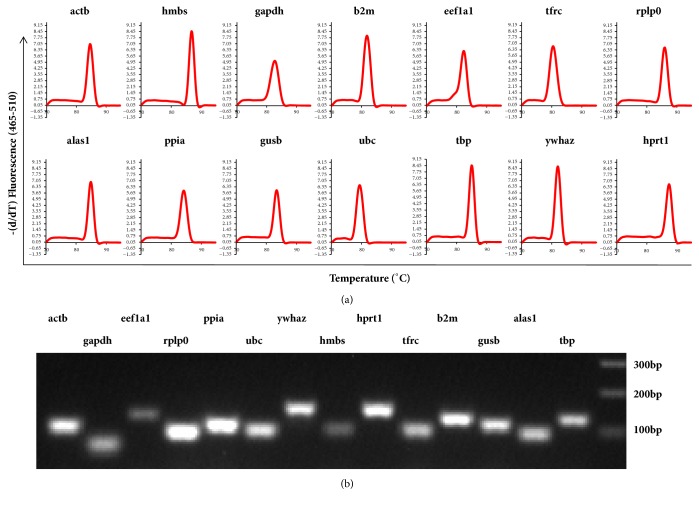
**Examination of primer specificity and size of RT-qPCR amplification productions.** (a) Melting curve analysis of the 14 reference genes amplicons after the RT-qPCR reactions. Only one peak for each primer was shown, suggesting high specificity of primers. (b) Examination of primer specificity and amplicon size. The high specificity with only one band as expected of each RT-qPCR amplification production using 2% agarose gel electrophoresis was shown.

Firstly, we identified expression level of the 14 RGs following the experimental procedure for qPCR using SYBR Green I Master [27]. The specificity of all the primers was evaluated using the melting curve with one single peak as shown in (Figure 1(a)). The PCR product and the band size for each primer were determined using 2% agarose gel electrophoresis. Gel imaging demonstrated that the size of all the amplified products was as expected; the bands were clear and there was no nonspecific banding as shown in Figure 1(b). In addition, there was no obvious primer dimer generation during PCR amplification with dH_2_O or RNA as template.

**Figure 2:**
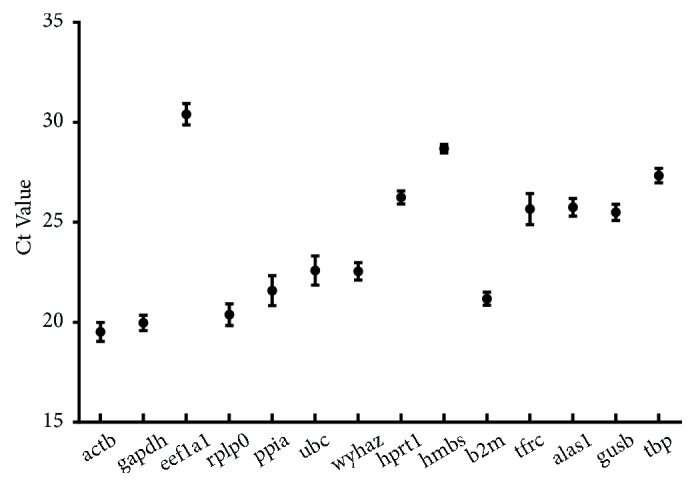
Mean Ct values of the 14 housekeeping genes in EC cells. Bars represent the mean ± standard deviation.

The primer pair efficiency ranged from 88.7% to 113% and coefficient (r^2^) values were between 0.989 and 1.0 as shown in Table 2. The expression levels of each gene were detected as Cp or CT value.  Figure 2 showed the different expression level between 19.9 and 30.4 cycles. actb showed the highest mRNA level while the mean Cp value (30.4) of eef1a1 expression level was lowest. Thus, it was not suitable as an internal reference gene.

### 3.2. Descriptive Statistic of Selected Reference Genes in Normal and Injured ECs

The RT-qPCR was used for the assessment of transcriptional expression of the 14 RGs in normal and injured ECs. The Cp values of all selected RGs using a threshold of 1.0 were detected except eef1a1 because of lowest expression level (missing Ct value in impaired endothelial cells). Evaluation of Cp values by statistical analysis revealed the variation and difference of RGs in normal and injured ECs. The 13 genes except eef1a1 were categorized into two groups based on the expression level. 7 genes (actb, gapdh, rplo, b_2_m, ppia, ubc, and ywhaz), whose Cp values fall between 21 and 24 cycles, are considered as the highly expressed genes. Those with Cp values between 28 and 26 cycles (gusb, alas1, tfrc, hrt1, hmbs, and tbp) were moderately expressed genes. In addition, the coefficient of variation (CV%) is shown in Table 3, with ywhaz (CV=9.96%), actb (CV= 9.23%), and alasi (CV= 9.16%) indicating the highest variation in gene expression, while ubc (CV= 6.65%) and tbp (4.32%) had the lowest one indicating the lowest variation in expression level of gene.

### 3.3. Examination of Reference Stability

#### 3.3.1. BestKeeper

The coefficient of variation (CV) expressed in percentage is used to determine the variation of the selected reference genes. The main factor in data analysis of CV: genes with the lowest value have lesser the variation [ubc (CV=6.65%); tbp (CV=4.32%)], while those with higher value [ywhaz (CV=9.66%); actb (CV=9.23%)] had more variation in gene expression. The Pearson coefficient of correlation (r^2^) is the index of the BestKeeper, which is used to determine the stability of the input reference gene and give the expression value. Genes with r^2^ closer to 1 are considered as the most stable genes; thus ywhaz and alas1 with r^2^ = 0.996 followed by actb (r^2^ = 0.978) are ranked as the highest stable genes, while ubc (r^2^ = 0.835) has the lowest value, indicating it had the lowest stability expression within these selected candidate reference genes as shown in Table 3.

#### 3.3.2. geNorm Analysis

**Figure 3:**
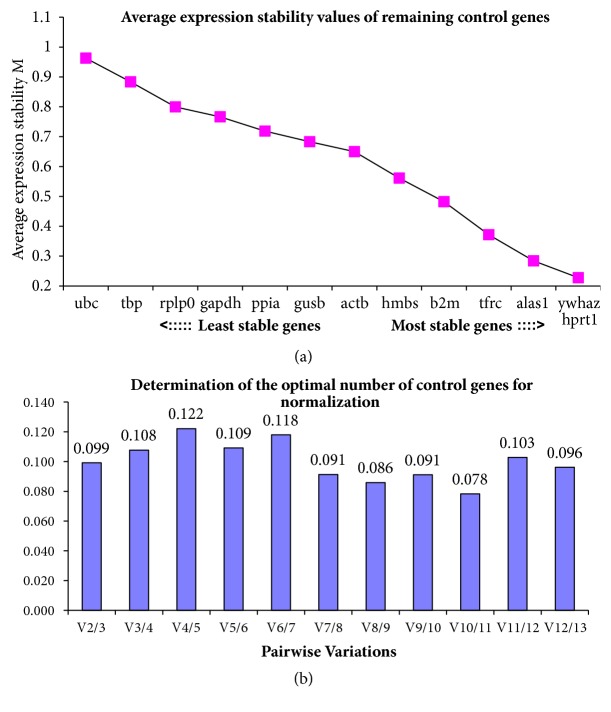
**Graphical presentation of stability value by geNorm**. (a) Showing the ranking of the 13 reference genes by geNorm software, with the most stable one toward the right and least stable one toward the left. (b) Determination of minimal number of reference genes by pairwise variation (Vn /n +1). The determination of optimal number of housekeeping genes by pairwise variation was shown. The effect of including more reference genes in a set of numbers of reference genes in all cases below the cut-off value of 0.15 is shown. The two most stable expressed reference genes may be accurate for qRT-PCR normalization. Including more reference genes for RT-qPCR normalization will not increase the stability of reference genes.

geNorm software grades the stability of the candidate genes based on the calculated M value. Genes with lower M value are considered as the most stable genes, while those with higher M value are the least stable genes. The three most stable genes include ywhaz and hprt1 both with an M value of 0.227 and alas1 with an M value of 0.284. The three reference genes with the highest M values were ubc (M = 0.962), tbp (M= 0.883), and rplo (M= 0.799), considered to be least stable expressed genes (Table 5 and Figure 3(a)). In addition to the geNorm software, pairwise variation was also used to determine the optimal number of candidate genes. All pairwise variation in this study was shown in Figure 3(b). According to [24], a cut-off value of 0.15 is recommended, where genes with a V ≤ 0.15 should be included because they indicate the lowest variation of reference gene normalization. Therefore, based on the cut-off value of 0.15, the two most stable reference genes (V_2_/V_3)_ of this dataset would be adequate for accurate normalization as shown in Figure 3(a).

#### 3.3.3. NormFinder

**Figure 4:**
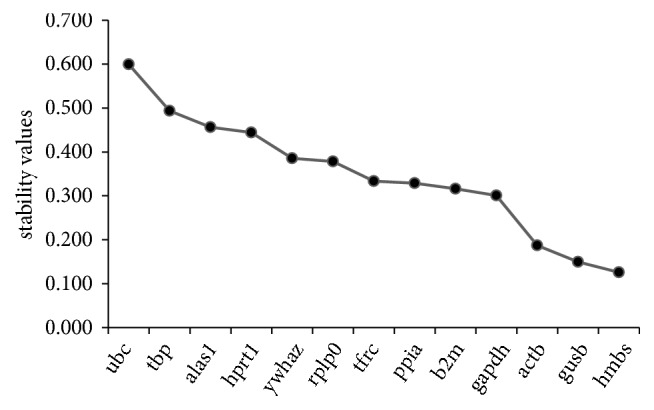
**Stability ranking values of reference genes by NormFinder**. It shows the stability ranking values of reference genes by NormFinder with the most stable gene being hmbs (0.126) and least stable gene being ubc (=0.599). The software also identified the best two combination genes, hmbs and gusb with a stability value of 0.081.

This software determined the stability value of the selected references and at the end it identified the best two combinations of genes. Genes with the lowest stability values are considered as the most stable (hmbs= 0.126, gusb=0.15, and actb=0.187), while those with the higher stability values are the least stable genes (ubc=0.599, tbc=0.494, and alas1= 0.456). The two best combined genes revealed by the software include hmbs and gusb with a stability value of 0.081 as shown in Figure 4.

#### 3.3.4. The Comparative ΔΔ CT

The ΔΔ** CT** method determines the stability of housekeeping genes based on relative expression comparison within the samples. The lower ΔCp value between samples of gene tested is considered to remain constant. In order to obtain the most stable gene, the lower ΔCp value is deducted from other Cp values [43, 44]. The data got from this method resemble those results we obtained from the BestKeeper. It ranked the alas1 and hprt1 as the two most stable RGs as shown in Table 4. In contrast to the geNorm software analysis, it identified ywhaz and hprt 1 as the most stable genes, while the NormFinder recognized hmbs as the most stable gene.

It illustrated the calculations of standard deviation (SD), variance, and mean fold difference using the delta-delta method and true fold difference determined by using log base 2 power of delta-delta value to determine the actual fold difference between the selected reference genes.

#### 3.3.5. Cumulative Ranking of All the Methods

We coranked the four methods to determine the most stable RGs in our study. In order to achieve the overall ranking, we allocate a weight to all the genes, which is then used to calculate the geometric mean [25]. The geometric mean value we obtained ranked ywhaz, alas1, and hmbs as the three most stable ones and gapdh, tbp, and ubc were considered as the least stable housekeeping genes as shown in Table 5.

#### 3.3.6. Illustration of the Impact of the Selected Housekeeping Genes on Normalized Fold Change of the Target Genes

**Figure 5:**
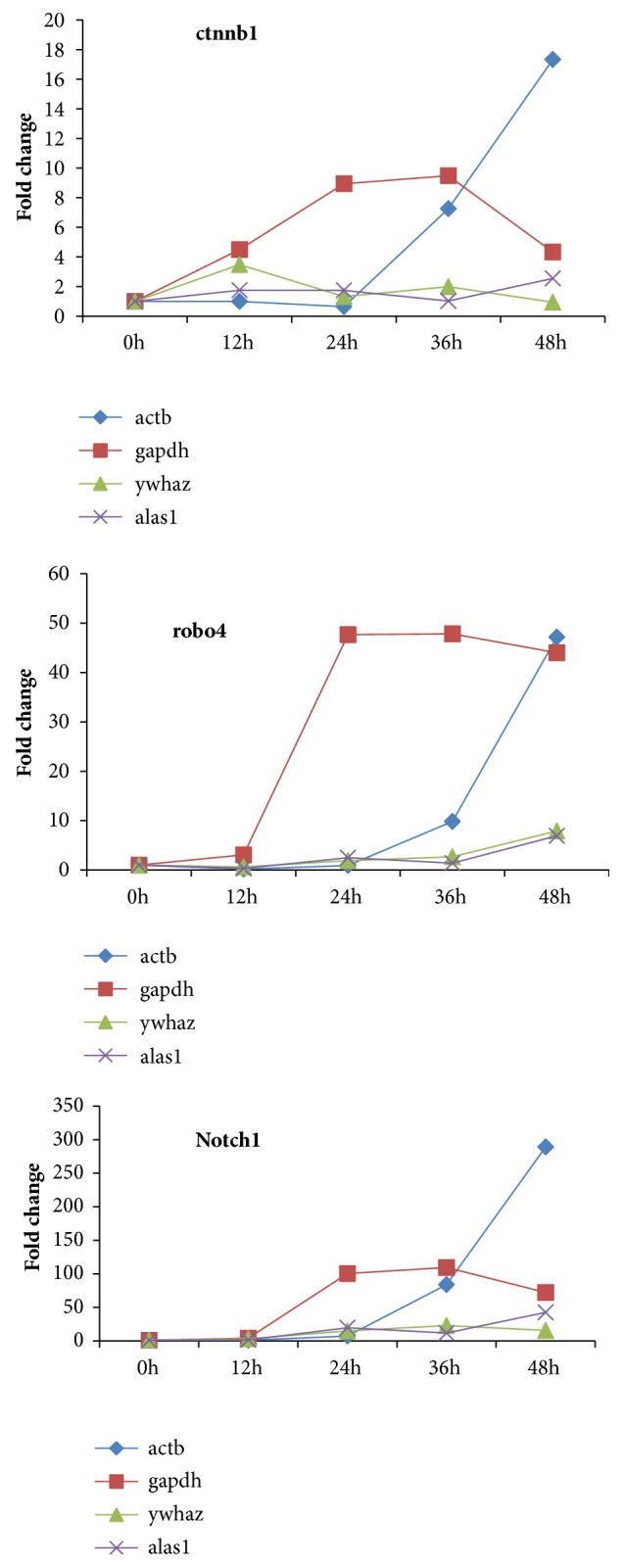
**The impact of the selected different housekeeping genes on the target gene expression**. Fold change values of three genes (ctnnb1, robo4, and notch1) were calculated based on actb, gapdh, ywhaz, and alas1. When using ywhaz and alas1 as reference genes, which were selected as the best two candidate housekeeping genes, the fold change values did not greatly increase over time. When using actb and gapdh as reference genes, which were commonly used, very higher values of fold change were obtained, with strong variation during cell injury.

To evaluate the performance of the selected reference genes, we detect three target genes from three different signalling pathways and normalized the gene expression level or the fold change values using different selected references genes. We selected the best two candidate genes (ywhaz and alas1) and the two most often used RGs (actb and gapdh). The results are shown in Figure 5, which suggested the strong variation when different reference genes were selected. When using ywhaz and alas1 as reference genes, which were selected as the best two candidate housekeeping genes, the fold change values did not greatly increase over time. When using actb and gapdh as reference genes, which were commonly used, very higher values of fold change were obtained, with strong variation during cell injury, which suggested actb and gapdh were not suitable as reference genes during gene expression analysis in Busulfan-impaired ECs.

#### 3.3.7. Effect of Busulfan on robo4 Expression in ECs

**Figure 6:**
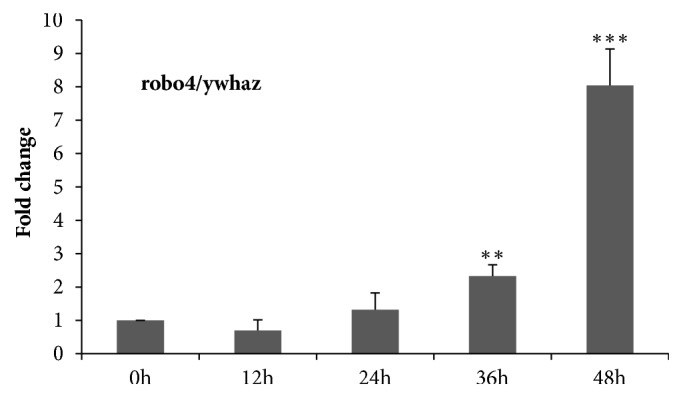
**Expression levels of the robo4 gene**. The normal and Busulfan-injured endothelial cells were used, using wyhaz, the best validated reference gene for normalization. Data are mean ±SEM, n =3;  ^*∗∗*^P< 0.01;  ^*∗∗∗*^P< 0.001 vs. 0h calculated from the 3 independent replicates.

When ywhaz was used as reference gene, we also found that robo4 expression level was significantly increased from 12h (0.7±0.3) to 48h (8.0±1.0) as shown in Figure 6.

## 4. Discussion

The ECs have been suggested recently to play an active role in normal hematopoiesis and HSC trafficking [6, 8]. Meanwhile, these cells are vulnerable to Busulfan, one bifunctional DNA alkylating agent widely used in preparative regimens in conditioning therapy for HSCT [1, 2]. Up to now, how the biomolecular expression changed in the injured ECs and how these injured ECs affected HSC are still unknown. Therefore, it is really important for screening of differential expression gene firstly, which will provide important information on the gene expression change in normal and Busulfan-injured ECs, especially those genes associated with HSC survival, homing, and trafficking. Gene expression changes caused by this drug can be detected accurately and sensitively by RT-qPCR. Several reports have ascribed that there is no specific housekeeping gene that can remain unchanged in all experiments and it is recommended that multiple genes can be used for normalization, in order to determine the most stable gene among the group of genes tested [45–47]. The concepts to verify and identify the housekeeping gene for normalization in RT-qPCR data analysis and to determine their stability under different experimental condition have been demonstrated [48–51]. The method seems to be easy, but normalization of data before use still remains an issue and needs to be validated. At present, there are still various studies regarding the identification of suitable HKGs by RT-qPCR. But, based on our knowledge, this is the first study of stable RGs selection in normal and Busulfan-injured ECs. In all, 14 RGs (actb, hmbs, hprt1, alas1, gapdh, gusb, ywhaz, rplo, ppia, tfrc, b2m, tbp, eef1a1, and ubc) were investigated in this study. The Ct value is usually used to determine the mRNA level by RT-qPCR data analysis. The same amount of RNA is used to obtain the gene expression levels. Generally, Cp value greater than 30 or lower than 15 is not suitable for RT-qPCR [52, 53]. In this research, the Ct values of selected RGs showed moderate variation in our samples. The Ct values of actb, gapdh, rplo, ppia, b2m, and ubc ranged from 21 to 23, while gusb and ywhaz ranged from 24 to 26 and the remaining reference genes alas1, tfrc, and tbp ranged from 27 to 28, respectively. Eef1a1 had the lowest expression level greater than 30, not suitable as RGs. Five different statistical methods previously reported [15] were used to compare the expression stability of these 13 candidate RGs. Based on the BestKeeper software analysis, the coefficient of variation, expressed in percentage, showed the variation in gene expression; thus genes with the highest CV values (ywhaz, actb, and alas 1) in sequential order had the highest variation in gene expression, in contrast to those with the lowest CV values (tbp, ubc, and hmbs), respectively. In addition, the Pearson correlation coefficient (r) closer to 1 represented the most stable gene. Based on the Pearson coefficient (r), ywhaz and alas1 were identified as the highest stability genes followed by hmbs, whereas ubc displayed the lowest stability. Our result revealed that actb was relatively stable and expressed in both normal and injured ECs in contrast to gapdh which was unstable. However, ywhaz, alasi, and hmbs were the three reference genes reported to be more stable in contrast to the gapdh, tbp, and ubc, which were the three least stable RGs. In accordance with previous studies, they identified HPRT1 and YWHAZ as the two most suitable genes in HUVEC-stain treated cells stimulated with TNF-*α* in relation to gene expression [25]. However, there was complete discrepancy in two recent studies, which identified TFRC, RPLO, GAPDH, and ACTB as the most stable genes [12] and the other studies found that hmbs, ywhaz, and tbp were considered as three most stable RGs in MSCs before and after differentiation [54]. Interestingly, in our study, ywhaz was ranked 1^st^, hmbs 3^rd^, actb 7^th^, tfrc 8^th^, gapdh11^th^, and rplo 9^th^, respectively. These findings suggested that it is very important to assess the expression stability of the selected RGs in any experimental research for ECs.

In our study we employed five methods (geNorm, NormFinder, BestKeeper, 2^-ΔΔCt^, and Comprehensive Ranking methods). The pairwise comparison approach includes the BestKeeper and geNorm, and this software identifies the most stable RG based on the expression of variation ratios among the RGs in our sample collected. The ratio variation (pairwise variation) for two candidate RGs across the sample measures the stability of the gene. However, the stability measurements of all the methods were combined to obtain the overall ranking of selected genes. The grading of RGs stability may be different due to the discrepancies of the program software. For example, the geNorm software ranked ywhaz and hprt 1 as the two most stable genes, while the NormFinder ranked the hmbs and gusb as the most stable genes. On the other hand, the BestKeeper ranked ywhaz and actb as the two most stable genes, while 2^-ΔΔCt^ method ranked alasi and hprt1 as the two most stable genes. So, finally, an overall ranking is established by calculating the geometric mean in order to minimize the differences between the statistical programs (geNorm, NormFinder and BestKeeper) and delta-delta method and have a final consensus of the most stable RGs. Based on the overall ranking, ywhaz was graded as the most stable among the 13 HKGs, together with alas1 and hmbs, respectively. These three genes were considered as the most appropriate HKGs because they displayed minimal fluctuations under our experimental conditions.

Some previous reports have suggested that accurate measurement of expression levels of normalization required multiple HKGs [24]. Here we performed RT-qPCR to test the mRNA expression level of three target genes based on the best two candidate genes (ywhaz and alas1) and the two most often used RGs (actb and gapdh) for normalization. Interestingly, we got very different results, which suggest that it is really an important thing to select suitable RGs before RT-qPCR was performed. The improper use of RGs would lead to a misinterpretation of gene expression data. But unfortunately, there were still a lot of researchers who did not provide a clear evidence for RGs selection. To our best knowledge, this is the first time to report the RG selection in Busulfan-injured ECs. Henceforth, our results will be very useful in guiding us/other researchers for further gene expression studies of ECs and encourage more investigators to perform RGs selection before gene expression analysis.

## 5. Conclusion

Our study demonstrated that Busulfan can strongly influence the stability of RGs. ywhaz and alas1 but not actb or gapdh were recognized as the idlest RGs for normal and injured ECs. This will be helpful for detection of gene expression in relation to both normal and Busulfan-injured ECs. Moreover, we also found that robo4 was significantly upregulated in Busulfan-injured ECs with time prolonged by optimized gene expression analysis. In conclusion, the results showed strong difference in gene expression level of three target genes using four different RGs which means that RGs selection is necessary for RT-qPCR normalization.

## Figures and Tables

**Table 1:** **Summary of 14 housekeeping genes and target genes evaluated in this study**. Official full name, accession numbers, official full name, primer sequences, and product sizes are shown.

**Symbol**	**Official full name**	**Physiological functions**	**Accession numbers**	**Primer sequence (forward/reverse)**	**Products size (bp)**
**gapdh**	Glyceraldehyde-3-phosphate dehydrogenase	An enzyme that catalyzed the sixth step of glycolysis, a process in which glucose is converted to pyruvate.	NM_001001303	F:catggccttccgtgttccta R:gcggcacgtcagatcca	55 [28–30]
**actb **	Beta-actin	Protein plays a key role in cell motility and cytoskeletal maintenance i.e. the structure and integrity.	NM_007393	F:atgtggatcagcaagcagga R:aagggtgtaaaacgcagctca	99 [31]
**ubc**	Ubiquitin C	Protein coded genes are involved in DNA repair and cell cycle regulation.	NM_019639.4	F:ccagtgttaccaccaagaag R:acccaagaacaagcacaagg	94
**eef1a1 **	eukaryotic translation elongation factor 1 alpha 1	Translation elongation factor	NM_010106	F:tccgattacgacgatgttga R:agtcgccttggacgttctt	125 [32]
**b2m**	Beta -2 microglobulin	This gene encodes serum protein found on MHC class I on the surface of all nucleated cells.	NM_009735	F:ttcagtatgttcggcttccc R:tggtgcttgtctcactgacc	103 [33, 34]
**rplp0**	Ribosomal protein lateral stalk subunit P0	It is a neutral phosphoprotein at the C-terminal end of ribosomal phosphoproteins	NM_007475	F:ccgatctgcagacacacact R:accctgaagtgctcgacatc	91 [35]
**ywhaz**	tyrosine 3-monooxygenase/tryptophan 5-monooxygenase activation protein, zeta	A central hub protein for many signal transduction pathways and is a major regulator of apoptotic pathways.	NM_011740	F:ctttctggttgcgaagcatt R:ttgagcagaagacggaaggt	148
**hmbs**	hydroxymethylbilane synthase	Providing instruction for making the enzyme hydroxymethylbilane synthase.	NM_013551	F:cagggtacaaggctttcagc R:cggagtcatgtccggtaac	149 [36]
**gusb**	*β*-glucuronidase	Providing instruction for producing an enzyme called beta- glucuronidase.	NM_010368	F:actcctcactgaacatgcga R:ataagacgcatcagaagccg	96 [37]
**ppia**	Peptidyl prolyl isomerase A	Cyclosporin binding protein /Inhibitor of serine threonine phosphatase	NM_008907	F:cagtgctcagagctcgaaagt R:gtgttcttcgacatcacggc	109 [38]
**tbp**	TATA box binding protein	Providing an instruction for making TAXA box binding proteins.	NM_013684	F:ggggtcataggagtcattgg R:catctcagcaacccacacag	127 [39]
**alas1**	*δ*-Aminolevulinate synthase 1	catalyzing the first step of heme biosynthesis	NM_020559	F:gtctgtgccatctgggactc R:ctgtccacatcagctgtcca	119
**hprt1**	Hypoxanthine phosphoribosyltransferase 1	providing instructions for producing an enzyme called hypoxanthine phosphoribosyl transferase 1	NM_013556	F:cataacctggttcatcatcgc R:tcctcctcagaccgctttt	95 [40]
**tfrc**	transferrin receptor	This gene encodes a cell surface receptor necessary for cellular iron uptake by the process of receptor-mediated endocytosis.	NM_011638	F:gcaccaacagctccaaagtc R:ccagtgtgggaacaggtctt	133 [41]
**ctnnb1**	catenin (cadherin associated protein), beta 1	The key function of this protein is to mediate the canonical Wnt signaling pathway, regulate gene transcription and mediate cell-cell adhesion	NM_007614.3	F: gtgcgctgagcttcaggt R: tcagctcgtgtcctgtgaag	147
**robo4**	Roundabout guidance receptor 4	Robo4 is a vascular-specific receptor.	NM_028783.3	F:cagcctggttagctcttctgatg R:gcacgagcaaagtgagtatcagc	57 [42]
**notch1**	Notch homolog 1	It plays a role in a variety of developmental processes by controlling cell fate decisions	NM_008714.3	F:ttcgtgctcctgttctttgtg R:gggctctctccgcttcttc	129

**Table 2:** Assay performance characteristics showing PCR coefficient (R^2^), slope, and primer pair efficiency (E).

**Gene name**	**Coefficient (R** ^**2**^ **)**	**slope (A)**	**primer pair efficiency (E)**
actb	1	-3.482	0.937
gapdh	0.99	-3.402	0.968
eef1a1	0.997	-3.282	1.017
rplp0	0.999	-0.325	0.999
ppia	0.999	-0.452	0.948
ubc	0.999	-0.365	0.982
ywhaz	1	-3.37	0.906
hprt1	1	-3.625	0.887
hmbs	0.996	-3.272	1.021
b2m	0.998	-3.405	1.021
tfrc	1.0	-3.215	0.966
alas1	0.989	-3.502	1.047
gusb	0.992	-3.042	0.93
tbp	1	-3.37	1.131

**Table 3:** Mean Ct, STD, coefficient of variation, and Pearson coefficient of candidates' RGs by BestKeeper.

**Gene names**	**Geomean**	**Ar Mean**	**Min [CP]**	**Max [CP]**	**STD [±CP]**	**CV **%	**Pearson coefficient [** **R** ^∧^ **2]**	**p-value**
ywhaz	24.33	24.51	22.17	32.58	2.37	9.66	0.996	0.001
actb	20.97	21.11	19.06	26.98	1.95	9.23	0.978	0.001
alas1	27.71	27.9	25.42	36.32	2.55	9.16	0.996	0.001
b2m	22.82	22.97	20.81	30.07	2.02	8.8	0.982	0.001
tfrc	27.15	27.32	25.2	35.58	2.29	8.4	0.99	0.001
ppia	23.15	23.27	20.73	28.96	1.92	8.26	0.962	0.001
hprt1	28.1	28.27	25.89	36.58	2.33	8.24	0.99	0.001
gapdh	21.13	21.23	19.62	26.18	1.7	8	0.958	0.001
rplpo	21.41	21.5	19.86	26.52	1.54	7.14	0.992	0.001
gusb	26.79	26.89	25.23	32.66	1.89	7.01	0.984	0.001
hmbs	28.83	28.94	26.98	35.4	2.03	7	0.994	0.001
ubc	23.13	23.24	21.04	29.42	1.59	6.85	0.835	0.002
tbp	28.15	28.2	26.93	32.47	1.22	4.32	0.96	0.001

**Table 4:** Expression stability detected by the comparative CT method (ΔΔCT method).

**Gene names**	**Mean Ct value**	**SD**	**Variance**	ΔΔ**Ct±SD**	**Mean fold difference **2^∧^-ΔΔ**CT**	**True fold difference log base**2^∧^-ΔΔ**Ct**
**actb**	21.105	2.672	7.139	-2.05±2.67	1.54±0.038	0.622
**gapdh**	21.348	2.085	4.97	-1.70±2.08	1.31±0.073	0.387
**rplo**	21.499	2.534	4.773	-1.65±2.53	1.45±0.055	0.540
**ppia**	23.373	2.534	6.423	-1.82±2.53	1.64±0.049	0.711
**ubc**	23.24	2.584	6.68	-2.21±2.58	1.31±0.036	0.379
**ywhaz**	24.512	3.462	11.986	-2.35±3.46	2.17±0.018	1.115
**hprt1**	28.266	3.519	12.383	-2.38±3.52	2.20±0.017	1.138
**hmbs**	28.939	2.842	8.077	-1.96±2.84	1.84±0.036	0.878
**b2m**	22.97	3.02	9.123	-2.16±3.02	1.82±0.028	0.86
**tfrc**	27.322	3.484	12.136	-2.13±3.48	1.33±0.020	0.408
**Alas1**	27.896	3.656	13.365	-2.48±3.66	2.26±0.014	1.175
**gusb**	26.894	2.587	6.693	-1.66±2.59	1.90±0.053	0.923
**tbp**	28.198	1.831	3.352	-1.27±1.83	1.47±0.012	0.558

**Table 5:** ** Ranking of thirteen RGs obtained using five different algorithms: ywhaz, **alas1, and hmbs were ranked as the most stable housekeeping genes while ubc, tbp, and gapdh were ranked as the least stable ones.

**Gene names**	**Bestkeeper**		**GeNorm**		**NormFinder**		2^∧^-ΔΔ**CT**		**Comprehensive Ranking**	
	**Pearson coefficient**	**ranking**	**Stability Values**	**ranking**	**Stability values**	**ranking**	**Mean fold difference**	**ranking**	**Geometric mean**	**Final ranking**
ywhaz	0.996	1	0.228	1	0.386	9	2.166	3	2.28	1
alas1	0.996	1	0.284	3	0.456	11	2.26	1	2.40	2
hmbs	0.994	3	0.561	6	0.126	1	1.838	5	3.08	3
hprt1	0.99	5	0.228	1	0.444	10	2.20	2	3.16	4
gusb	0.984	7	0.683	8	0.15	2	1.896	4	4.60	5
b2m	0.982	8	0.482	5	0.316	5	1.815	6	5.89	6
actb	0.978	9	0.650	7	0.187	3	1.540	8	6.24	7
tfrc	0.99	5	0.372	4	0.333	7	1.327	11	6.26	8
rplo	0.992	4	0.799	11	0.378	8	1.454	10	7.70	9
ppia	0.962	10	0.719	9	0.329	6	1.637	7	7.84	10
gapdh	0.958	12	0.766	10	0.301	4	1.308	12	8.71	11
tbp	0.96	11	0.883	12	0.494	12	1.472	9	10.93	12
ubc	0.835	13	0.963	13	0.599	13	1.30	13	13.00	13

## Data Availability

The data used to support the findings of this study are included within the article.
